# Loss of G-Protein Pathway Suppressor 2 Promotes Tumor Growth Through Activation of AKT Signaling

**DOI:** 10.3389/fcell.2020.608044

**Published:** 2021-01-07

**Authors:** Stefanie Chan, Emma Smith, Yuan Gao, Julian Kwan, Benjamin C. Blum, Andrew M. Tilston-Lunel, Isabella Turcinovic, Xaralabos Varelas, Maria Dafne Cardamone, Stefano Monti, Andrew Emili, Valentina Perissi

**Affiliations:** ^1^Department of Biochemistry, Boston University School of Medicine, Boston, MA, United States; ^2^Center for Network Systems Biology, Boston University, Boston, MA, United States; ^3^Division of Computational Biology, Department of Medicine, Boston University School of Medicine, Boston, MA, United States

**Keywords:** breast cancer, GPS2, ubiquitin (Ub), MK2206 (PubChem CID: 46930998), Akt

## Abstract

G Protein Suppressor 2 (GPS2) is a multifunctional protein that exerts important roles in inflammation and metabolism in adipose, liver, and immune cells. GPS2 has recently been identified as a significantly mutated gene in breast cancer and other malignancies and proposed to work as a putative tumor suppressor. However, molecular mechanisms by which GPS2 prevents cancer development and/or progression are largely unknown. Here, we have profiled the phenotypic changes induced by GPS2 depletion in MDA-MB-231 triple negative breast cancer cells and investigated the underlying molecular mechanisms. We found that GPS2-deleted MDA-MB-231 cells exhibited increased proliferative, migratory, and invasive properties *in vitro*, and conferred greater tumor burden *in vivo* in an orthotopic xenograft mouse model. Transcriptomic, proteomic and phospho-proteomic profiling of GPS2-deleted MBA-MB-231 revealed a network of altered signals that relate to cell growth and PI3K/AKT signaling. Overlay of GPS2-regulated gene expression with MDA-MB-231 cells modified to express constitutively active AKT showed significant overlap, suggesting that sustained AKT activation is associated with loss of GPS2. Accordingly, we demonstrate that the pro-oncogenic phenotypes associated with GPS2 deletion are rescued by pharmacological inhibition of AKT with MK2206. Collectively, these observations confirm a tumor suppressor role for GPS2 and reveal that loss of GPS2 promotes breast cancer cell proliferation and tumor growth through uncontrolled activation of AKT signaling. Moreover, our study points to GPS2 as a potential biomarker for a subclass of breast cancers that would be responsive to PI3K-class inhibitor drugs.

## Introduction

Comprising nearly a third of all newly diagnosed cancers, breast cancer is the most common cancer affecting women worldwide. Overall mortality from breast cancer has declined greatly in the past decades due to better screening and improved treatment options. However, the absence of effective treatments for metastatic breast cancers, together with the risk of recurrence due to intrinsic or acquired resistance to endocrine therapies and the poor prognosis of triple negative breast cancers remain critical challenges. In the attempt of identifying novel targetable nodes and mutational signatures that could inform patient-specific, targeted therapeutic approaches, multiple large scale studies have characterized the genomic alterations and mutational burden associated with primary and metastatic breast cancers (Koboldt et al., 2012; Nik-Zainal et al., 2016; Martincorena et al., 2017). Among others, mutation of the *GPS2* (G-protein Pathway Suppressor 2) gene was identified as a potential driver in both settings, in agreement with similar observations reported from genomic profiling of other cancer cohorts, including pan-cancer metastatic solid tumors (Priestley et al., 2019) and medulloblastomas (Pugh et al., 2012).

G Protein Suppressor 2 is a small, ubiquitous protein originally identified as a suppressor of Ras activation in the yeast pheromone response pathway (Spain et al., 1996) and an inhibitor of JNK activity in mammalian cells (Jin et al., 1997; Zhang et al., 2002). Emerging evidences indicate that GPS2 downregulation plays an important role in the development of obesity and associated metabolic disorders, through altered regulation of inflammation, mitochondria biogenesis, and lipid metabolism in a variety of cell types, including adipose, liver, and immune cells (Cardamone et al., 2012, 2018; Toubal et al., 2013; Fan et al., 2016; Cederquist et al., 2017; Drareni et al., 2018; Liang et al., 2019). GPS2 has also been reported to function as a tumor suppressor in liposarcoma (Huang et al., 2016), in agreement with predictive analyses from genome sequencing data (Kumar et al., 2015). However, a mechanistic understanding of GPS2 role in cancer is currently lacking.

In normal cells, GPS2 actions are exerted through a combination of genomic and non-genomic functions based on the shared mechanism of GPS2 inhibiting non-proteolytic ubiquitination mediated by Ube2N/Ubc13 (Cardamone et al., 2012, 2014, 2018; Cederquist et al., 2017; Lentucci et al., 2017). Ubiquitination is a reversible modification that is achieved via the sequential actions of several classes of enzymes, including an ubiquitin (Ub)-activating enzyme (E1), an Ub-conjugating enzyme (E2), and an Ub ligase (E3). Poly-ubiquitination of target proteins with chains of different topology can either promote protein degradation or modulate protein functions and interactions through non-proteolytic signals (Komander, 2009). A key player for the formation of non-proteolytic, K63-linked ubiquitin chains is the E2 conjugating enzyme Ube2N/Ubc13, which works in complex with non-catalytic subunits mms2/Uev1A and specific E3 ligases (Hodge et al., 2016; Stewart et al., 2016). GPS2 binds to and inhibits the activity of Ubc13 in the context of pro-inflammatory signaling pathways, chromatin remodeling events, and insulin signaling (Cardamone et al., 2012, 2014, 2018; Cederquist et al., 2017; Lentucci et al., 2017). In the case of insulin signaling, GPS2-mediated inhibition of Ubc13 in adipocytes was found necessary to prevent uncontrolled ubiquitination and activation of the Protein Kinase B (PKB, also known as AKT) (Cederquist et al., 2017).

The serine/threonine kinase AKT is one of the most frequently activated oncoprotein in human cancer, with its critical role in breast cancer tumorigenesis and tumor development being supported by extensive studies in human cancers and mouse models (Fruman et al., 2017; Hoxhaj and Manning, 2020). Tumor cells can achieve AKT activation through a variety of mechanism, including not only its own amplification but also the functional activation of upstream positive regulators, like PI3K kinase, or inactivation of negative regulators, like PTEN (Manning and Cantley, 2007; Manning and Toker, 2017). In normal cells, full AKT activation is promoted by translocation to the plasma membrane and dual phosphorylation by PDK1, a PI3K-dependent kinase, and mTORC2, a component of the mTOR complex (Manning and Toker, 2017). Recent evidences indicate that non-proteolytic ubiquitination of AKT, and possibly other factors, provide an additional level of regulation for the PI3K/AKT/mTOR pathway, with K63 ubiquitination of AKT favoring its recruitment to the membrane, and thus activation (Yang et al., 2009, 2010; Fan et al., 2013; Linares et al., 2013; Guo and Wei, 2019; Wang et al., 2019). Relevance of this regulatory strategy is supported by the existence of a specific cancer-associated mutation (AKT E17K) that displays enhanced ubiquitination and constitutive membrane localization (Fan et al., 2013; Wang et al., 2019), and by the observation that reactivation of AKT through Skp2-mediated ubiquitination plays a role in promoting resistance to PI3K inhibitors in TNBC (Clement et al., 2018).

Here, we have investigated GPS2 role as a tumor suppressor in the context of triple negative breast cancer and provide evidences that loss of GPS2 promotes cell proliferation and tumor growth through sustained AKT activation.

## Materials and Methods

### Cell Culture

MDA-MB231 and MDA-MB468 cells were maintained in DMEM with 4.5 g/L glucose and L-glutamine and 10% FBS at 37°C and 5% CO_2_.

### Generation of Cell Lines

#### GPS2-KO Lines

For each cell line studied (MDA-MB-231 and MDA-MB-468) we generated two independent KO lines (KO1 and KO2) by CRISPR-Cas9 genome editing using two different sets of sgRNAs. In each case, sgRNAs targeted exons 2 and 6 of the human GPS2 sequence. The two sets of sgRNAs each targeting were cloned separately into the LentiCRISPRv2 lentiviral vector (Kindly shared by Dr. Feng Zhang, Addgene plasmid #52961). 3.5 mg of each viral plasmid was co-transfected along with packaging plasmids pCMV-VSV-G and ps.PAX2 (Addgene plasmids #8454 and #12260) into HEK293T cells using Lipofectamine 3000. Media was changed after 24 hours and viral supernatant collected at 60 h. The virus was filtered through a 0.46 μm low protein binding filter membrane and either used immediately or stored at -80°C for later use. Transduction of target cell lines (MDA-MB-231 and MDA-MB-468) was performed in a six-well plate with 1–1.5 ml of viral supernatant and 8μg/ml polybrene in DMEM with 10% FBS. Media was changed after 24 h and cells split into selection media (puromycin 2.5 μg/ml) at 48 h. The KO lines were maintained as heterogeneous pools without selection of individual clones.

#### MB231-WT and -GPS2KO RFP Lines

Red Fluorescent Protein (RFP)-expressing lentivirus was prepared as the GPS2-KO lentivirus, using the LV-RFP vector (Kindly shared by Dr. Elaine Fuchs, Addgene plasmid # 26001). RFP positive cells were FACS sorted 48 h after infection.

#### MB231-myrAKT Line

myrAKT retrovirus was made by co-transfecting HEK293T cells with 2.8 mg pUMVC, 0.2 mg VSV-G, and 2 mg pBABEpuro-myrFLAG-AKT (Kindly shared by Dr. William Hahn, Addgene plasmid # 15294) using Lipofectamine3000. Media was changed after 24 h and viral supernatant collected at 60 h.

### Protein Isolation and Western Blotting

Whole cell extracts prepared by homogenization in IPH lysis buffer (50 mM Tris–HCl pH 8, 250 mM NaCl, 5 mM EDTA, 0.5% NP-40) supplemented with 0.1 mM PMSF, 2 mM NaV, 50 mM NaF, and 1× protease inhibitors (Sigma Aldrich). Cells were resuspended in lysis buffer for 20 min on ice then spun at maximum speed for 10 min at 4°C. Supernatant was collected and protein concentration quantified via Bradford assay against BSA standards (Bio-Rad) and normalized prior to loading. Extracts were boiled with NuPAGE LDS sample buffer and 100 mM DTT for 5 min at 95°C prior to loading on 10% Mini-PROTEAN TGX gels (Bio-Rad) in SDS-PAGE running buffer (25 mM Tris, 192 mM glycine, 0.1% SDS) (Boston Bioproducts) at 180 V for 1 h. Proteins were transferred onto 0.45 μm low fluorescent PVDF membranes (Bio-Rad) using the TransBlot Turbo Transfer System (Bio-Rad). Membranes were blocked in either 5% non-fat milk or 5% BSA in PBST (137 mM NaCl,5.4 mM KCl, 16.2mM Na_2_HPO_4_, 2.9 mM KH_2_PO_4_, 0.1% Tween-20) for one hour at room temperature (RT) before overnight incubation with primary antibody at 4°C. The next day membranes were washed 3 × 10 min in PBST and incubated in appropriate fluorescent secondary antibody (Bio-Rad) and tubulin conjugated to hFAB Rhodamine for 1 h at RT, washed again 3 × 10 min in PBST and visualized with the ChemiDoc MP Imaging System and Image Lab Software (Bio-Rad). Proteins were compared to the Precision Plus Protein Standard protein ladder (Bio-Rad) for sizing.

### Cell-Based Functional Assays

#### Scratch Assay

About 1.5 × 10^6^ cells were seeded in a six-well plate, upon attachment a 200 μl pipette tip was used to scratch the cell monolayer. Cell debris was gently washed away and fresh media with or without 10 μM MK2206 was added. Pictures were taken immediately after insult (0 h) and at specified timepoints (7 and 24 h).

#### Proliferation Assay

Cells were seeded 2 × 10^4^ (MB231) or 5 × 10^4^ (MB468) per well in a 24-well plate with or without 10 μM MK2206 and incubated at 37°C. At each 24-h timepoint media was aspirated and cells were washed with 1×PBS, then fixed in cold methanol for 20 min at −20°C. Cells were then briefly stained with 2.3% crystal violet (Sigma) and rinsed with water until color no longer came off in rinse. Crystal violet was then solubilized in 10% glacial acetic acid and quantified at 590 nm on a plate reader.

#### Transwell Migration Assay

After overnight starvation, cells were seeded 5 × 10^4^ in 300 μl serum free media in the upper chamber of an 8 μm transwell with or without 10 μM MK2206. 500 μl of complete media was added to the lower chamber and after 24 h of incubation at 37°C the transwell insert was removed and washed with 1×PBS, and apical surface of insert scrubbed with a cotton swab. Cells on the basal surface were then fixed with cold methanol and stained with crystal violet as described above. Crystal violet was solubilized and quantified as described above.

#### Matrigel Invasion Assay

Matrigel inserts were rehydrated in serum free media for 2 h at 37°C, then the top chamber seeded with 5 × 10^4^ serum starved cells in 300 μl serum free media with or without 10 μM MK2206 while 500 μl of complete media was added to the lower chamber. After 24 h of incubation at 37°C the Matrigel insert was removed and processed as described for transwell inserts.

### Animal Studies

Six-week-old NOD *scid* gamma (NSG) mice (NOD.Cg-PrkdcscidIl2rg^TM 1*Wjl*^/SzJ, stock #005557) were ordered from Jackson Laboratory. Mice were kept on a standard laboratory chow diet and a 12-h light/dark cycle. All studies were approved by the Boston University Institutional Animal Care and Use Committee (IACUC).

#### Orthotopic Xenograft Model of MB231-GPS2KO

About 2 × 10^6^ MB231-WT or MB231-GPS2KO cells were injected into the 4th mammary fat pad of 6 to 8-week-old female NSG mice. Once palpable tumors formed, tumors were measured once weekly by IVIS imaging or twice a week by caliper. For manual measurements, tumor volume was calculated as V = (W(2) × L)/2. Once tumors reached a volume of 200 mm^3^, mice were weighed and dosed weekly with either 360 mg/kg MK2206 or 30% Captisol by oral gavage.

#### *In vivo* Imaging

The Xenogen IVIS Spectrum Instrument and Living Image Software Version 3.2 (Caliper LifeSciences) was used for imaging, analysis, and quantification of fluorescent RFP signal. Up to five mice at a time were anesthetized (1–3% isoflurane), placed in a warmed imaging chamber with continuous isoflurane exposure (1–2% isoflurane), and imaged for up to 1 min once a week.

### Histology

Samples were fixed in 10% formalin and submitted to BU Experimental Pathology Laboratory Core for paraffin embedding and sectioning. Immunohistochemistry (IHC) was performed on paraffin sections. Sections were deparaffinized and cleared with 3 × 5-min washes in Histo-Clear (National Diagnostics), followed by the following EtOH washes: 2 × 5 min 100%, 2 min 95%, 1 min each of 85, 70, 50%. Sections were then washed in dH_2_O for 2 min. For antigen retrieval, citrate buffer (10 mM citric acid, 0.05% Tween 20, pH 6.0) was heated to 95–100°C, and slides were microwaved for 10 min on low power (30%) to unmask epitopes and immediately quenched in tap water. Slides were then incubated in fresh 3% hydrogen peroxide on shaker at RT for 15 min, and washed with PBS (pH 7.4) 2 × 5 min. Sections were outlined with ImmEdge hydrophobic barrier pen, blocked in 5% donkey serum in PBS for 45 min, and incubated in primary antibody diluted in 5% donkey serum/PBS in a humid chamber overnight at 4°C. After quick rinsing in PBS and TBST, sections were washed at RT for 4 × 10 min in TBST while shaking. Next, sections were washed 2 × 5 min in PBS, incubated in SignalStain Boost IHC Detection Reagent (Cell Signaling) at RT for 45 min, then washed 3 × 10 min in TBST. SignalStain DAB substrate kit (Cell Signaling) was applied until brown stain was visible, and reaction was immediately quenched in water. Sections were counterstained for 6 min in Harris hematoxylin, then washed for 10 min under running tap water, followed by a dip in acid alcohol (70% EtOH, 1% HCl), then Scott’s tap water. Sections were dehydrated by the following washes: EtOH 30 s 50%, 80%, 2 × 2 min 100%, 5 min 100%, Histo-Clear 3 × 5min. Sections were then mounted with CystoSeal XYL (Thermo Fisher Scientific) and coverslips for imaging.

### RNA-Seq

Total RNA was harvested from cultured cells using Qiagen RNeasy Plus Mini Kit according to manufacturer’s instructions. Triplicates of each condition were sent to BU’s Microarray and Sequencing core for library preparation using Kapa RNA HyperPrep with RiboErase and run on two high output 75-paired end sequencing runs on Illumina NextSeq.

#### RNAseq Data Processing

Sequenced data was processed based on the RNAseq pipeline implemented in *Pipeliner* (Federico et al., 2019). In particular, sequencing reads were checked for quality with *FastQC* (Wingett and Andrews, 2018), trimmed with *TrimGalore* (Krueger), then aligned to the reference genome hg38 with *HISAT2* (Kim et al., 2015), and quantified with *featureCounts* (Liao et al., 2014). After alignment, mapping quality was checked with RSeQC (Wang et al., 2012), and a comprehensive summary report of all samples was generated with MultiQC (Wang et al., 2012).

#### Gene Expression Analysis

Gene expression differential analysis was performed for each of the manipulations of interest based on DEseq2 (Love et al., 2014). The expression matrix was first filtered so as to remove genes with less than one read per million in at least three samples. Pairwise differential analyses were then performed for the following conditions (3 replicates/condition): MB231-GPS2KO (KO) vs. MB231-WT (WT); MB231-myrAKT (AKT) vs. WT; KO vs. MB231-GPS2 KO + MK. Nominal *p*-values were corrected for multiple hypothesis testing by the FDR procedure (Benjamini and Hochberg, 1995). Signatures were defined for each condition as the set of up- and down-regulated genes with *q*-value ≤0.05 and fold change ≥1.5 in either direction.

#### Signature Projection and Survival Analysis

The derived differential analysis signatures were then used to analyze the TCGA breast cancer dataset by signature projection. In particular, patient-specific signature projection scores were computed by *Gene Set Variation Analysis (GSVA;* Hänzelmann et al., 2013) for each of the up- and down-regulated signatures of interest. Furthermore, a summary score for each condition was computed by taking the difference between the up-regulated and down-regulated signature scores. For example, to compute the signature projection scores of GPS2KO *vs.* WT, the corresponding up- and down-regulated signatures were input to GSVA, and *gps.up* and *gps.dn* scores were computed for each of the TCGA BRCA subjects. A summary *gps* score was computed as *gps* = *gps.up – gps.dn* for each of the subjects. Survival analyses for each of the conditions of interest were performed based on a log-rank test and Kaplan Meyer curves comparing subject groups obtained by stratifying subjects based on their signature scores being greater than or lower than the median score value for a given condition signature.

### Proteomic and Phosphoproteomic Studies

#### Protein Digestion

After pelleting, the protein precipitate resulting from organic solvent extraction (protein crash) was resuspended in 100 μl of 8M urea buffer containing protease inhibitors (Sigma) and phosphatase inhibitors (Roche). After brief sonication on ice, the samples were reduced by addition of dithiothreitol (DTT) to a final concentration of 5 mM for 60 min at RT, and alkylated by the addition of iodoacetamide (5 mM) and incubation at RT for 30 min in the dark. Proteins were diluted with 50 mM ammonium bicarbonate to bring urea concentration down to below 1M and digested with sequence-grade trypsin (1:50 enzyme to protein ratio) at 37°C overnight followed by the addition of formic acid to 1% in solution. The resulting peptides were desalted using C18 Tips per manufacturer’s instructions (Thermo Fisher Scientific).

#### TMT Peptide Labeling

Peptide quantification was determined by Pierce quantitative colorimetric assay (Thermo Fisher Scientific) and 100 μg of peptide per sample was resuspended in 0.1 M triethylammonium bicarbonate (TEAB) and incubated with the TMT 10-plex isobaric label reagents (0.8 mg Thermo Fisher Scientific). The ratio of TMT to substrate was 0.4 mg regent to 0.1 mg peptide. Reaction was carried out for 1 h at room temperature. To quench the reaction, 5% hydroxylamine was added to each sample and incubated for 15 min. Equal amounts of each sample were combined in a new tube and a speed vac was used to dry the labeled peptide sample. The labeled peptides were desalted using C18 Tips (Thermo Fisher Scientific).

#### High pH Reverse Phase Fractionation of Peptides

Peptides (1 mg) were fractionated offline on a Waters XBridge BEH C18 (3.5 μm, 4.6 × 250 mm) reverse phase column using an Agilent 1100 HPLC system operated at a flow rate of 0.45 ml/min with two buffer lines: buffer A (consisting of 0.1% ammonium hydroxide-2% acetonitrile-water) and buffer B (consisting of 0.1% ammonium hydroxide-98% acetonitrile, pH 9). Peptides were separated by a gradient from 0 to 10% B in 5 min, followed by a linear increase to 30% B in 23 min, 60% B in 7 min, and then 100% in 8 min and continued for 5 min, resulting in 48 fractions that were collected but were combined into 12 fractions prior to being evaporated to dryness in a vacuum concentrator. 2 μg of peptide from each fraction was reconstituted in 1% formic acid, and kept in −80°C prior to analysis by nLC-MS/MS.

#### Titanium Dioxide (TiO_2_) Enrichment of Fractionated Phosphopeptides

Titanium dioxide coated magnetic beads were used to enrich phosphopeptides obtained from HPLC fractions. Beads were pre-incubated in a DHB buffer (5 μl/mg) consisting of 6% TFA, 5 mM KH_2_PO_4_, 80% ACN, 20 mg/ml 2,5-dyhydroxybenzoic acid for 15 min. The peptide mixture from each fraction was resuspended in 500 μl of DHB buffer and incubated with beads (10:1 bead to peptide ratio, w/w) for 30 min while shaking. Beads were step washed: 1% TFA-80% ACN, followed by 1% TFA-50% ACN, and 1% TFA-10% ACN twice, then the supernatant was discarded. The bound phosphopeptides were eluted by 5% NH4OH with 25% ACN and dried by speed vac before nLC-MS analysis.

#### Nanoflow LC-MS of Proteomics and Phosphoproteomics

Both peptides obtained from the HPLC fractionated sample digests and the bead-enriched phosphopeptides from each fraction were individually loaded onto a C18 trap column (3 μm, 75μm × 2 cm, Thermo Fisher Scientific) connected in-line to a C18 analytical column (2 μm, 75 μm × 50 cm, Thermo EasySpray) using the Thermo EasyLC 1200 system with the column oven set to 55°C. The nanoflow gradient consisted of buffer A (composed of 2% (v/v) ACN with 0.1% formic acid) and buffer B (consisting of 80% (v/v) ACN with 0.1% formic acid). For protein analysis, nLC was performed for 180 min at a flow rate of 250 nl/min, with a gradient of 2–8% B for 5 min, followed by a 8–20% B for 96 min, a 20–35% gradient for 56 min, and a 35–98% B gradient for 3 min, 98% buffer B for 3 min, 100-0% gradient of B for 3 min, and finishing with 5% B for 14 min. Peptides were directly ionized using a nanospray ion source into a Q-Exactive HF mass spectrometer (Thermo Fisher Scientific).

The QE-HF was run using data dependent MS2 scan mode, with the top 10 most intense ions acquired per profile mode full-scan precursor mass spectrum subject to HCD fragmentation. Full MS spectra were collected at a resolution of 120,000 with an AGC of 3e6 or maximum injection time of 60 ms and a scan range of 350–1650 m/z, while the MS2 scans were performed at 45,000 resolution, with an ion-packet setting of 2e4 for AGC, maximum injection time of 90 ms, and using 33% NCE. Source ionization parameters were optimized with the spray voltage at 2.1 kV, transfer temperature at 275°C. Dynamic exclusion was set to 40 s.

For phosphoprotein analysis, the enriched phosphopeptides were loaded onto a 50 cm C18 microcolumn followed by separation over a 90 min gradient using a flow rate of 250 nl/min, with a gradient of 2–6% B for 5 min, followed by a 6–20% B for 39 min, a 20–35% gradient for 23 min, and a 35–98% B gradient for 3 min, 98% buffer B for 3 min, 100–0% gradient of B for 3 min, and finishing with 5% B for 14 min. In this case, data dependent MS2 scans were performed on the top six ions, with the maximum injection time of MS2 of 400 ms.

#### Analysis of Raw Mass Spectrometry Proteomic Data

All acquired MS/MS spectra were searched against the Uniprot human complete proteome FASTA database downloaded on 2018_10_26, using the MaxQuant software (Version 1.6.7.0) that integrates the Andromeda search engine. TMT reporter ion quantification was performed using MaxQuant. Enzyme specificity was set to trypsin and up to two missed cleavages were allowed. Cysteine carbamidomethylation was specified as a fixed modification whereas oxidation of methionine and N-terminal protein acetylation were set as variable modifications. For phosphopeptides serine, threonine, and tyrosine phosphorylation were specified as variable modifications. Peptide precursor ions were searched with a maximum mass deviation of 6 ppm and fragment ions with a maximum mass deviation of 20 ppm. Peptide and protein identifications were filtered at 1% FDR using the target-decoy database search strategy. Proteins that could not be differentiated based on MS/MS spectra alone were grouped to protein groups (default MaxQuant settings). A threshold Andromeda score of 40 and a threshold delta score of 8 was applied to phosphopeptides, in accordance with parameters described previously. The MaxQuant output files designated “Phospho(STY)sites” and “ProteinGroups” were used for data normalization and other statistical analysis using in-house generated scripts in the R environment.

#### Data Analysis and Pathway Enrichment

Bioinformatic analysis was performed using R: A language and environment for Statistical Computing (R Foundation for Statistical Computing, Vienna, Austria) version 3.6.1. The MaxQuant tables of protein group and phosphosite feature intensities was log transformed and loess normalization was applied. For differential analysis, the LIMMA (Ritchie et al., 2015) R package was used to fit a linear model accounting for the experimental conditions. Moderated t-tests were corrected with the Benjamini-Hochberg method for false discovery rate (FDR). Gene set enrichment analysis was performed using the fgsea R package (Sergushichev, 2016) using gene libraries generated by the Bader lab (Bader et al., 2003).

## Results

### MDA-MB-231 GPS2-KO Transcriptional Profiling

**FIGURE 1:**
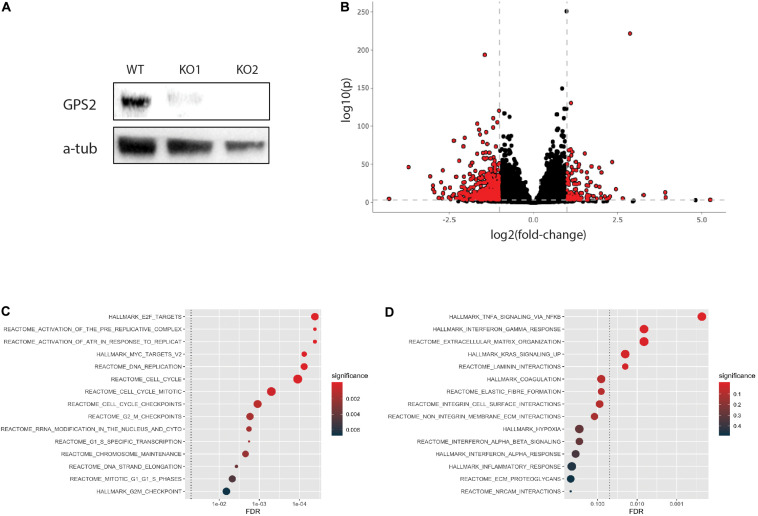
Generation and transcriptomic profiling of MB231-GPS2KO cell lines. **(A)** Western blot (WB) for GPS2 on whole cell lysate (WCL) from MDA-MB-231 WT and GPS2KO cells. **(B)** Volcano plot of DEGs between WT and MB231-GPS2KO cells(FDR ≤ 0.001, Log2FC ≥ 1). **(C)** HypeR-enrichment analysis of upregulated genes in MB231-GPS2KO cells as compared to the parental line. **(D)** Federico and Monti (2020) of downregulated genes in MB231-GPS2KO cells as compared to the parental line.

To begin investigating the function of GPS2 as a tumor suppressor in triple negative breast cancer, we generated two MDA-MB-231-GPS2 knockout lines by CRISPR-Cas9 genome editing with two independent sets of sgRNA (Supplementary Figure 1A). Efficacy of gene knockdown was confirmed by western blot (Figure 1A) and genomic PCR (Supplementary Figure 1B). We then compared the transcriptome of both MDA-MB-231-GPS2KO cell lines (from here on called MB231-GPS2KO) to the parental wild type MDA-MB-231 cells by RNA-Seq. Using a threshold of FDR ≤0.001 and fold change ≥1.5, we identified a total of 2081 differentially expressed genes (DEG), including 649 upregulated and 1432 downregulated genes, between WT and KO cells with significant correlation observed across the two KO lines (Figure 1B, Supplementary Figure 1C, and Supplementary Table 1). Pathway analysis on upregulated genes showed significant enrichment for terms associated with cell cycle, mitotic checkpoints, MYC and E2F transcription, and DNA replication (Figure 1C), all which suggest an increase in proliferation. Down regulated genes were enriched for terms associated with integrins, laminins and extracellular matrix (ECM) interaction (Figure 1D), pointing toward a possible increase in cell mobility and invasion potential due to reduced association with the ECM.

### Loss of GPS2 Leads to Increased Proliferation and Migration of MDA-MB-231 Cells

**FIGURE 2:**
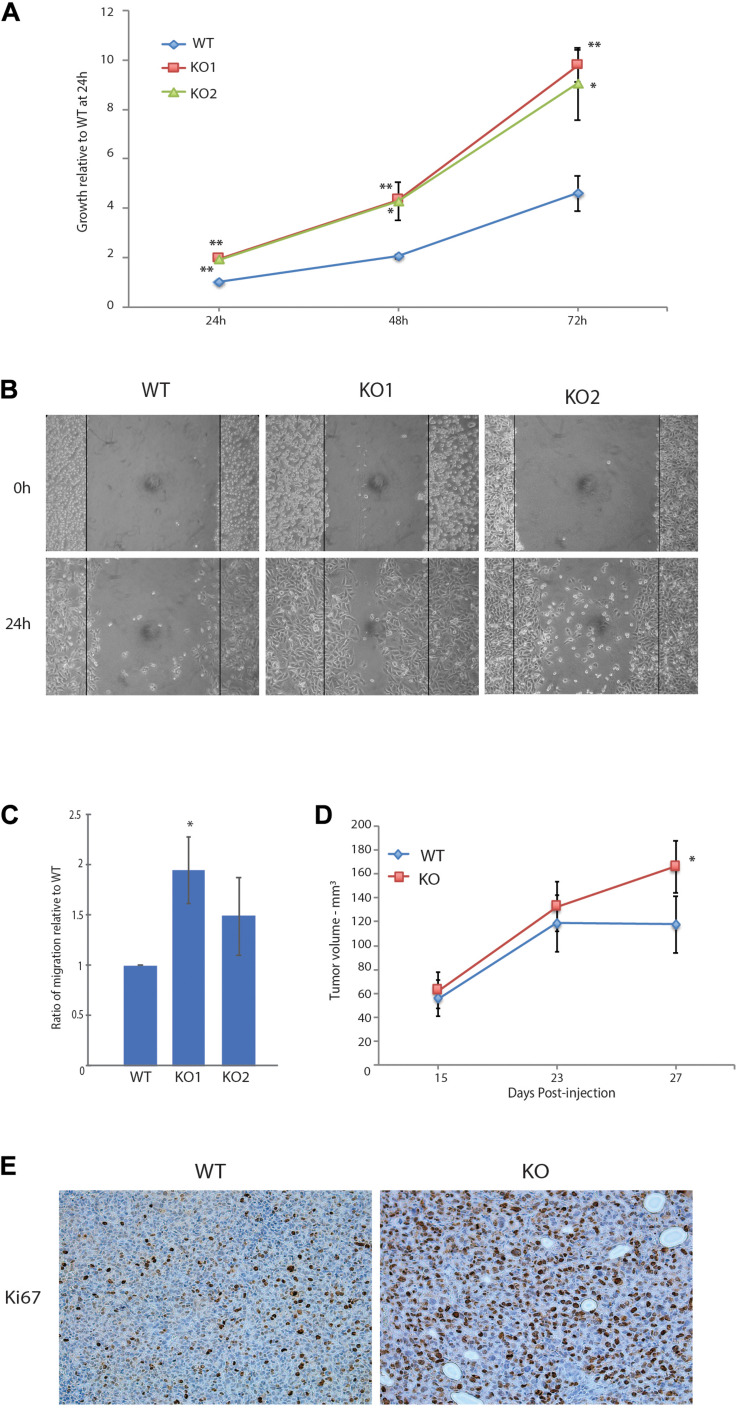
Loss of GPS2 leads to increased proliferation and migration in MDA-MB-231. **(A)** 72-h proliferation assay with MB231-WT and -GPS2KO cell lines. Cells were seeded 2 × 10^4^ per well in a 24-well plate, and fixed and stained with crystal violet every 24 h. **(B)** 24-h scratch assay of MB231-WT and -GPS2KO cell lines. Cells were seeded 1.5 × 106 per well of a 6 well plate and imaged 0h and 24h after scratch induction. **(C)** Transwell migration assay of MB231-WT and -GPS2KO cell lines. Cells were seeded 5 × 10^4^ in the upper chamber in serum free medium. After 24 h, cells that migrated to full medium chamber below were fixed, stained and quantified with crystal violet. **(D)** Tumor volume as measured by caliper and calculated via the equation V = (W(2)xL)/2. N = 10 mice/group. **(E)** Representative Ki67 staining of harvested MB231-WT and -GPS2KO primary tumors. Graphs of cell-based assays represent the mean ± SEM of at least three replicate experiments performed with technical triplicates. **p < 0.005, *p < 0.05 as compared to WT.

We next characterized the phenotypic changes associated with the loss of GPS2 through cell-based assays. In accord with the pathway enrichment analysis, MB231-GPS2KO cell lines showed a marked increase in their proliferation rate, doubling almost twice as much as the parental control line in the first 24-h period (Figure 2A). A 24-h scratch assay measuring migration of cells across a defined gap showed that MB231-GPS2KO cells were also more efficient at wound closure in comparison to the parental WT line (Figure 2B). Similar results were observed with acute downregulation of GPS2 by transient siRNA transfection (Supplementary Figure 2A). To remove the possibility of increased proliferation affecting the wound healing rate, wound closure was also measured at an earlier time point, prior to the first doubling time of 31 h (Risinger et al., 2015). MB231-GPS2KO cells performed similarly at 7 and 24 h (Supplementary Figure 2B) indicating it is unlikely that the observed increase in wound-healing rate is exclusively a byproduct of increased proliferation. To further measure the migratory properties of the MB231-GPS2KO cells, we performed a transwell migration assay. Over the course of 24 h, both MB231-GPS2KO lines appeared to migrate at a slightly higher rate than the WT even though the difference was statistically significant for only one of the KO lines (Figure 2C). These results, together, indicate that the loss of GPS2 promotes both proliferative and migratory capacity of MDA-MB231 cells *in vitro.*

We next sought to see if the effects seen *in vitro* could be replicated *in vivo*. A dual orthotopic xenograft breast cancer mouse model was developed by injection of MDA-MB-231 WT and GPS2KO cells in opposing mammary fat pads of NSG mice. All mice developed primary tumors on both sides, with GPS2 null cells leading to significantly enlarged tumor size (nearly 30%) in comparison to matched WT-derived tumors (Figure 2D). These results confirm a tumor suppressor role for GPS2 in breast cancer, in agreement with *in silico* analysis of the distribution of GPS2 expression across human tumor types, using data from the TCGA PanCancer Studies on cBioPortal, showing a trend toward low GPS2 expression, associated with deletions and truncating events, in invasive breast cancer samples (Supplementary Figure 2C). Moreover, IHC of sections from xenograft tumors showed increased levels of the proliferation marker Ki67 in MB231-GPS2KO tumors as compared to their WT counterparts (Figure 2E), thus indicating that the increased tumor burden in a breast cancer model of GPS2 depletion is due, at least partially, to increased proliferation of GPS2 null cells.

### Proteomic and Phosphoproteomic Profiling of MDA-MB231-GPS2KO Cells

**FIGURE 3:**
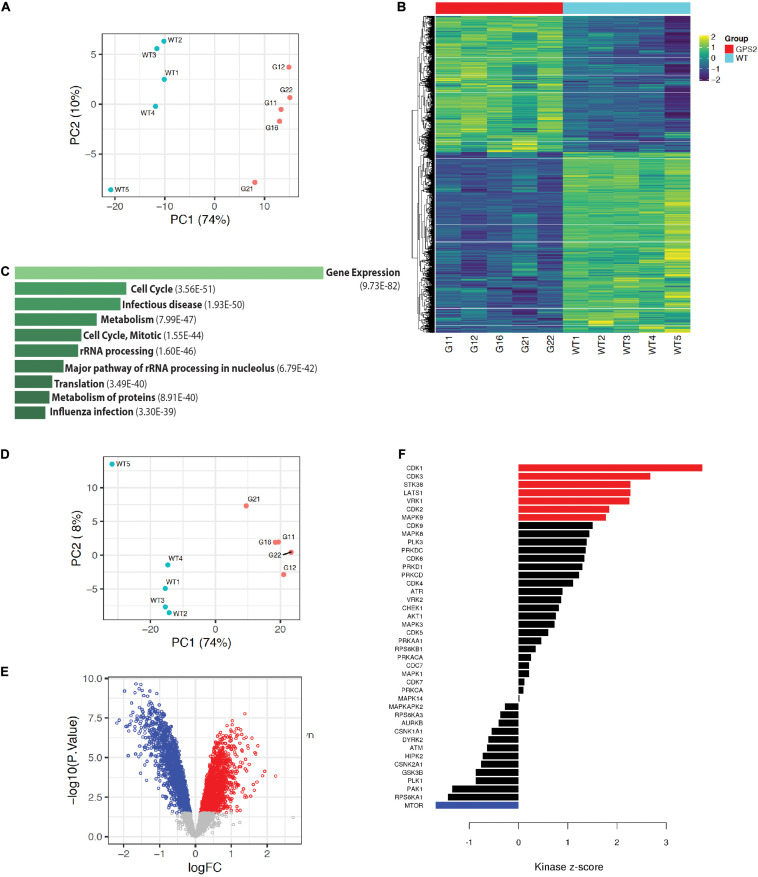
Proteomic profiling of MB231-GPS2KO cells. **(A)** PCA plot of proteomic MB231-WT and -GPS2KO datasets. **(B)** Heatmap of differentially expressed protein signatures across MB231-WT and -GPS2KO replicate samples. **(C)** Ten most significant pathways associated with differentially expressed proteins in MB231-GPS2KO cells as compared to the WT parental line based on the Reactome database (EnrichR). Bars represent Adjusted p value as indicated in parenthesis. **(D)** PCA plot of phosphoproteomic datasets from MB231-WT and -GPS2KO normalized to the corresponding proteomic data. **(E)** Volcano plot of up- and down-regulated phosphosites in MB231-GPS2KO cells as compared to WT cells. **(F)** Bar plot of Kinase Sites Enriched among up- and down-regulated phosphosites in MB231-GPS2KO as compared to WT cells (KSEA analysis).

To further characterize the effect of GPS2 depletion in MDA-MB-231 cells and investigate the underlying molecular mechanisms by which GPS2 acts as a tumor suppressor, we performed proteomic and phosphoproteomic profiling of five independent replicate samples of MDA-MB-231 WT and GPS2KO cells by LC-MS/MS, using isobaric tandem mass tag (TMT) labeling for relative quantification. As highlighted by principal component analysis, the two genotypes are characterized by distinct proteomic signatures with 4711 differentially expressed proteins across five replicates (FDR < 0.05), including 890 proteins upregulated and 1312 proteins downregulated (LOGFC > 0.25) in both GPS2KO lines as compared to the parental cells (Figures 3A,B and Supplementary Table 2). In agreement with the growth phenotype, differentially expressed proteins are enriched for terms associated with “cell cycle” and “translation”(Figure 3C). Also over-represented are proteins involved in metabolic pathways, in accord with previous reports of GPS2 regulating insulin signaling and lipid metabolism in adipose tissue (Cardamone et al., 2012, 2014, 2018; Cederquist et al., 2017; Drareni et al., 2018; Supplementary Figure 3A). IGF/AKT/mTOR signaling pathways, in particular, were found enriched among upregulated proteins, together with terms associated with antiapoptotic pathways, protein translation and lipid synthesis (Supplementary Figure 3B). Downregulated proteins instead were enriched for terms related to focal adhesion and ECM, including TGF beta, collagen binding and integrin signaling (Supplementary Figure 3C). In accord with the previously described role in regulating mitochondria-nuclear communication and transcription of nuclear-encoded mitochondrial genes, downregulated proteins also include a variety of mitochondrial proteins, including key metabolic enzymes, such as succinate dehydrogenase subunits SDHC and SDHD and succinate dehydrogenase assembly factor SDHAF3, isocitrate dehydrogenase IDH1 and several subunits of various electron transport chain (ETC) complexes.

Phosphoproteomic profiling similarly showed definite separation between WT and KO, as indicated by PCA analysis with or without normalization to the proteomic data set (Figure 3D and Supplementary Figure 3D). In total, we identified 3305 differentially regulated phosphosites on 1264 proteins, including 1681 downregulated and 1345 upregulated phosphosites (based on adj *p* value <0.05 and LOGFC >0.25) (Figure 3E and Supplementary Table 3). Concurrent with the increased proliferative phenotype, Kinase-Substrate Enrichment Analysis (KSEA) showed positive enrichment of cell cycle kinases (CDK1/2/3) (Figure 3F). Activity of MAPK9 (JNK) and AKT1 was also found positively enriched (Figure 3F), in agreement with previous studies on GPS2 depletion/downregulation in adipose tissue and immune cells (Cardamone et al., 2012; Lentucci et al., 2017).

### Loss of GPS2 Promotes Reprogramming of Gene Expression Through Constitutive AKT Activation

**FIGURE 4:**
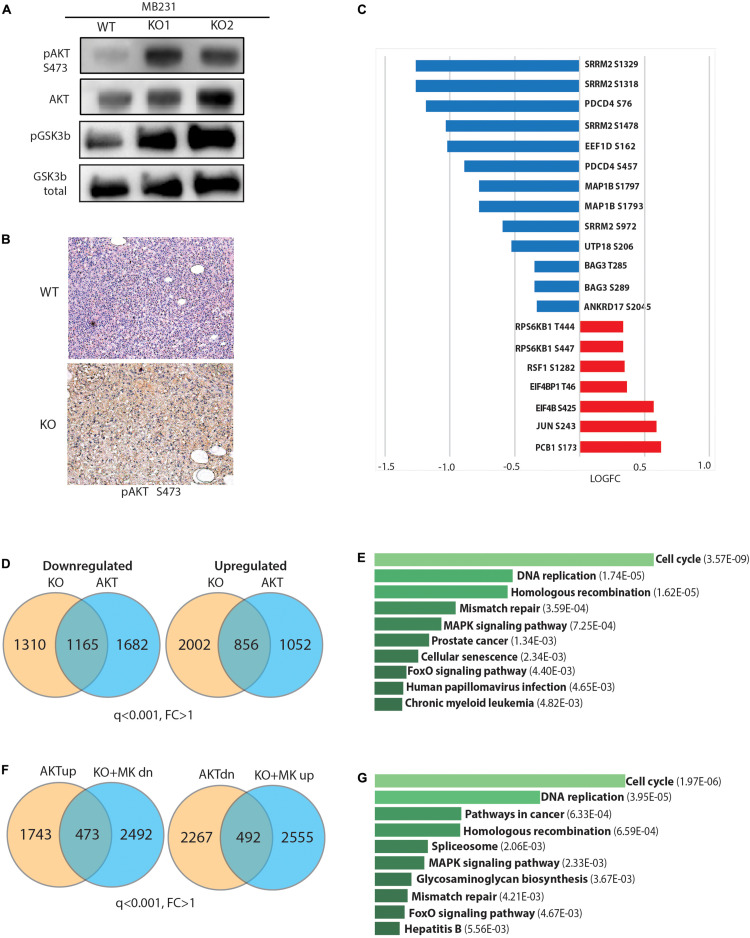
Loss of GPS2 results in increased AKT activity in cells with no prior PI3K/AKT pathway activation. **(A)** WB of whole cell extracts from MB231-WT and MB231-GPS2-KO cells showing pAKT(S473), total AKT, pGSK3b, total GSK3b. **(B)** Representative pAKT IHC staining of harvested MB231-WT and -GPS2KO tumors. **(C)** Changes in abundance of selected phosphosites in the MB231-GPS2KO phosphoproteomic signature. **(D)** Venn diagram showing overlap between DEG in MB231-GPS2KO and MB231-myrAKT. **(E)** Ten most significant pathways enriched among overlapping genes between MB231-GPS2KO and MB231-myrAKT gene signatures based on the human KEGG database (EnrichR). Bars represent Adjusted p value as indicated in parenthesis. **(F)** Overlay of genes upregulated in MB231-myrAKT cells with genes downregulated in MB231-GPS2KO upon treatment with MK2206, and downregulated genes in MB231-myrAKT with genes upregulated in MB231-GPS2KO upon treatment with MK2206. **(G)** Ten most significant pathways enriched among differentially expressed genes in MB231-myrAKT cells that are responsive to MK2206 treatment in MB231-GPS2KO based on the human KEGG database (EnrichR). Bars represent Adjusted p value as indicated in parenthesis.

We next focused on AKT regulation as a putative mechanistic explanation of the observed phenotypes. First, we tested whether GPS2 depletion was sufficient to promote the phosphorylation of AKT on Ser476, and found a dramatic increase in the basal levels of p-AKT in starved GPS2-deleted cells compared to WT (Figure 4A). In accord with our previous findings (Cederquist et al., 2017), constitutive AKT activation in absence of GPS2 is associated with an increase in non-proteolytic K63 ubiquitination as shown by IP/WB (Supplementary Figure 4A). IHC staining for pAKT in tumor samples collected from mice bearing orthotopic xenograft models also showed a significant increase in active AKT in tumors derived from MB231-GPS2KO cells as compared with matching WT tumors (Figure 4B). Together these results demonstrated that loss of GPS2 promotes activation of AKT kinase in breast cancer cells, *in vitro* and *in vivo*, similarly to what previously reported in adipocytes (Cederquist et al., 2017).

Next, we investigated the extent to which this results in activation of downstream signaling. By western blotting we observed increased phosphorylation of GSK3b, a key downstream effector of the PI3K/AKT signaling cascade (Figure 4A). We also overlaid the MB231-GPS2KO phosphoproteomic signature with known targets of mTOR and AGC kinases (Hsu et al., 2011; Yu et al., 2011). Most canonical targets were found upregulated in MB231-GPS2KO cells, including known phosphosites on EIF4b, 4E-BP1, S6K, and PRAS40 (Figure 4C and Supplementary Table 4), which confirms that GPS2 deletion leads to activation of classic pro-oncogenic signaling cascades downstream of AKT. However, there was also a number of targets that instead were found downregulated, in agreement with mTOR sites being enriched among downregulated protein in the KSEA analysis (Figure 3F).

To further address whether GPS2 depletion is sufficient to drive full activation of AKT-dependent signaling, we generated a new line of MDA-MB-231 cells expressing constitutively active, myristoylated AKT1 (MB231-myrAKT) and profiled them by RNA-Seq analysis (Supplementary Figure 4B). By using a threshold of q ≤ 0.001, we identified a total of 4975 DEG genes, including 2216 upregulated and 2759 downregulated genes (Supplementary Figure 4C and Supplementary Table 5). Overlay of this MDA-MB-231 specific AKT signature with that of MB231-GPS2KO cells confirmed a significant overlap of their transcriptomic profiles, with the two lines sharing approximately half of the downregulated genes and a quarter of the upregulated genes (Figure 4D and Supplementary Table 5). This included genes involved in classic pathways regulated by PI3K/AKT signaling, including “cell cycle,” “DNA replication,” and “FoxO signaling” (Figure 4E).

To further confirm that the altered expression of this large transcriptional program in response to GPS2 depletion is caused by aberrant activation of the AKT pathway, we attempted rescuing the expression of target genes by treating the MB231-GPS2KO cells with MK2206, an allosteric AKT inhibitor. Profiling by RNA-seq of MB231-GPS2KO cells treated for 4 h with 10 μM MK2206 revealed 1096 DEG genes (Supplementary Figures 4D,E and Supplementary Table 5). Overlay of these gene signatures confirmed the presence of a transcriptional program co-regulated in MB231-GPS2KO and MB231-myrAKT cells that is responsive to AKT inhibition, including 492 upregulated and 473 downregulated genes enriched for pathways involving “cell cycle,” “DNA replication,” “cancer,” “MAPK signaling,” and “FoxO signaling.” On the contrary, genes differentially regulated in MB231-GPS2KO cells but not altered in response to AKT activation were enriched for “Myc targets,” “RNA processing,” and “mitochondrial translation” when upregulated, and “ECM interactions,” “inflammatory responses,” and “lipid metabolism” among the downregulated genes (Figures 4F,G and Supplementary Table 6). Together, these results suggest that loss of GPS2 promotes cell proliferation at least partly through activation of AKT and downstream signaling, even though altered expression of additional pathways, such as activation of Myc-driven transcription, may contribute to the observed phenotypic outcomes.

### MDA-MB-231-GPS2KO Phenotype Is Responsive to AKT Pathway Inhibition

**FIGURE 5:**
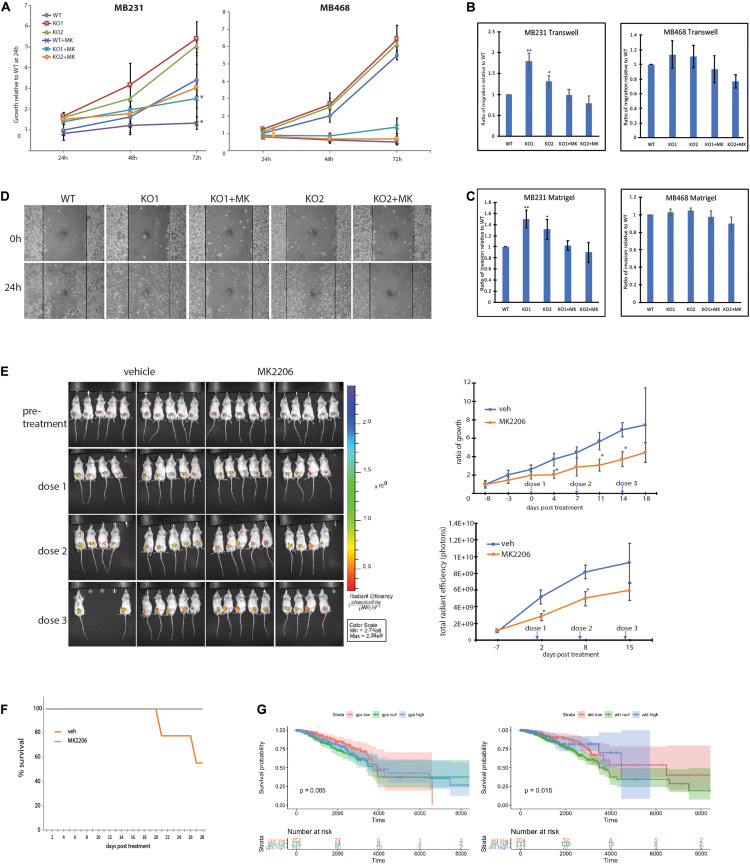
MB231-GPS2KO phenotype is responsive to AKT pathway inhibition. **(A)** 72-h proliferation assay of indicated cell lines in presence or absence of 10 μM MK2206. For a 24 well plate, MB231 were seeded 2 × 10^4^ per well and MB468 were seeded 5 × 10^4^ per well, each cell line was fixed and stained with crystal violet every 24 h. Graphs represent the mean of at least three replicate experiments ± SEM. *p < 0.05, when comparing non-treated lines to corresponding lines treated with MK2206. **(B)** Transwell migration assay of indicated cell lines in presence or absence of 10 μM MK2206. Cells were seeded 5 × 10^4^ in the upper chamber in serum free medium. After 24 h, cells that migrated to full medium chamber below were fixed, stained and quantified with crystal violet. **(C)** Matrigel invasion assay of indicated cell lines in presence or absence of 10 μM MK2206. Cells were seeded 5 × 10^4^ in the upper chamber in serum free medium. After 24 h, cells that invaded to full medium chamber below were fixed, stained and quantified with crystal violet. **(D)** 24-h scratch assay of MB231-WT and -GPS2KO cell lines in presence or absence of 10 μM MK2206. Cells were seeded 1.5 × 106 per well of a 6 well plate and imaged 0 and 24 h after scratch induction. **(E)** (Left): IVIS visualization of MB231-GPS2KO-RFP tumors in mice (*n* = 20) treated with either 360mg/kg MK2206 or 30% Captisol (vehicle) once per week for 3 weeks. Empty spaces correspond to mice that that died during the course of treatment or had to be sacrificed prior to the experimental endpoint due to excessive tumor burden. (Top Right): Ratio of tumor growth as measured by manual caliper. (Bottom Right): Quantification of total radian efficiency from IVIS measurements. **(F)** KM survival curve of mice bearing MB231-GPS2KO-RFP tumors, treated either with MK2206 or vehicle. **(G)** Stratification of survival of TCGA subjects based on MB231-GPS2KO and MB231-myrAKT gene signatures. Unless otherwise indicated, graphs represent the mean ± SEM of at least three replicate experiments performed with technical triplicates. **p < 0.005, *p < 0.05 as compared to WT. For mouse studies, *n* = 10 for each group.

To further investigate the extent to which GPS2 role as tumor suppressor depends on its ability to prevent aberrant activation of the PI3K/AKT pathway, we tested whether treatment with the allosteric AKT inhibitor MK2206 was sufficient to rescue the phenotypic changes associated with the loss of GPS2. As predicted on the basis of transcriptomic studies, the proliferation of MB231-GPS2KO was reduced to near WT levels upon treatment (Figure 5A). Similar results were observed in transwell and Matrigel assays, with the increased migration and invasion observed in MB231-GPS2KO cells as compared to the WT parental line being almost completely ablated by AKT inhibition (Figures 5B,C). Inhibition of AKT activity by MK2206 also reverted the increased wound healing rate of MB231-GPS2KO cells in a 24h scratch assay (Figure 5D).

In parallel to the analysis of MDA-MB-231 cells, we investigate the effect of GPS2 depletion by CRISPR-Cas9 genome editing on AKT activation and associated phenotypes in MDA-MB-468, a TNBC cell line characterized by constitutive activation of the PI3K/AKT pathway due to loss of the PI3K inhibitor PTEN. Contrary to MB231-GPS2KO, loss of GPS2 in MDA-MB-468 cells did not promote any further increase in AKT phosphorylation beyond the level of basal activation observed in the parental line (Supplementary Figure 5A). Accordingly, MB468-GPS2KO cells did not display significant changes in the proliferation rate or the migration/invasion assays when compared to WT parental cells (Figures 5A–C). Together these results indicate that the oncogenic phenotypic effects of GPS2 deletion observed in MDA-MB231 are largely due to activation of AKT through a mechanism that is alternative to the loss of PTEN-mediated inhibition.

To confirm the relevance of these findings *in vivo*, we tested the efficacy of inhibiting AKT activation in rescuing GPS2 deletion in our xenograft mouse model by injecting the mammary gland of 20 mice with MDA-MB-231-GPS2KO cells tagged with red fluorescent protein (MB231-GPS2KO-RFP) and treating half of them with weekly doses of 360mg/kg MK2206. As expected, mice treated with MK2206 had a relatively lower tumor burden over the 3-week treatment period, as measured both by manual caliper and quantification of total radian efficiency (photons) via IVIS (Figure 5E). Treatment with MK2206 also increased overall survival of mice bearing MDA-MB-231-GPS2KO-RFP tumors, with all mice treated with MK2206 surviving to the end of the experiment, as opposed to 55% of vehicle-treated mice (Figure 5F). In agreement with these results, projection of the GPS2KO and Myr-AKT gene signatures onto TCGA breast cancer survival curves revealed a marginal survival advantage associated with tumors with higher GPS2 expression/lower AKT activation (Figure 5G). While this result may be partially explained by the fact that low GPS2 activity is enriched in basal subtypes known to associate with poorer prognosis (Supplementary Figures 5B,C), it is worth noting that lower GPS2 expression in fact correlates with poorer survival in breast cancer patients (Supplemental Figure 5C; Györffy et al., 2010).

## Discussion

A comprehensive list of tumor suppressors and oncogenes altered in cancers is critical to fully understand what drives tumor growth and to identify molecular pathways and single proteins to target for therapeutic design. While specific mutations are often associated small number of cases, it is critical that large scale screenings are complemented with detailed molecular studies that provide mechanistic insights to drive patient-specific treatment choices. In this study, we have characterized the role of GPS2 in TNBC cells using a combination of *in vitro* and *in vivo* approaches. Overall, our results have confirmed that GPS2 functions as a tumor suppressor, as previously suggested by multiple evidences from genomic profiling of human cancer biopsies and by *in vitro* data from liposarcoma (Pugh et al., 2012; Huang et al., 2016; Priestley et al., 2019). Depletion of GPS2 in MDA-MB-231 cells in fact correlates with an increase in proliferation, migration and invasion *in vitro*, and increased tumor burden with elevated cell proliferation in an *in vivo* xenograft mouse model of breast cancer. Transcriptomic, proteomic and phosphoproteomic profiling of MDA-MB-231-WT and GPS2KO cells aligned with the observed phenotypes and confirmed that the loss of GPS2 in transformed cells promotes reprogramming toward an even more aggressive phenotype, even though it remains to be determined whether loss of GPS2 alone is sufficient to drive tumorigenesis and further studies are needed to investigate the effect of GPS2 deletion on metastasis formation.

Mechanistically, our results indicate that GPS2 deletion leads to constitutive activation of AKT signaling both *in vitro* and *in vivo*. Levels of pAKT were found increased in MB231-GPS2KO both by WB of cellular extracts and in tumor samples taken from orthotopic xenograft models, in agreement with the enrichment for AKT substrates observed by phospho-proteomic profiling and with previous findings from our lab showing that aberrant upregulation of AKT ubiquitination promoted sustained AKT signaling in GPS2 null adipocytes (Cederquist et al., 2017). Also, most importantly, our data indicate that the phenotypic changes observed upon GPS2 depletion are rescued by treatment with the AKT inhibitor MK2206. Collectively, these results provide a mechanistic explanation for the altered phenotype of GPS2KO cells and suggest that monitoring GPS2 expression level and mutational status may be useful for identifying tumors responsive to inhibition of the PI3K/AKT pathway. While this may be especially relevant for TNBC, an aggressive cancer subtype that currently lacks effective targeted therapies, we speculate that GPS2 status may be equally important in forecasting HR + tumor responsiveness to both PI3K/AKT inhibition and hormonal therapy via estrogen deprivation/anti-estrogen treatment. As a member of the NCoR/SMRT corepressor complex, GPS2 is thought to function as a transcriptional corepressor of ERα-mediated transcriptional regulation, with depletion of GPS2 compromising tamoxifen mediated repression (Cheng and Kao, 2009). One advantage of the TNBC model used in this study is that it allowed to explore the effects of GPS2 depletion on cytosolic signaling pathways without confounding results due to altered regulation by hormone receptors. However, in the context of ER-positive breast cancer, loss of GPS2 may correlate not only with upregulation of PI3K/AKT signaling but also with the development of endocrine resistance, similarly to what previously reported in association with reduced NCoR and SMRT expression (Wong et al., 2014). To address this possibility, further studies will be required to investigate the effects of GPS2 depletion in ER-positive cells and other cancer models where GPS2 transcriptional function may be more relevant. Comparisons across cell types will aid in dissecting the role played by GPS2 and non-proteolytic K63 ubiquitination in the crosstalk between AKT, hormone receptors signaling and possibly other pro-oncogenic signaling pathways. As Ubc13 overexpression was reported to promote breast cancer metastasis through aberrant activation of a TAK1/p38 MAPK kinase cascade (Wu et al., 2014), it is possible, for example, that loss of GPS2 regulates cell proliferation and migration through complementary pathways. Even in the context of primary tumor growth, misregulation of other pathways may be a contributing factor as indicated by our overlay of MB231-GPS2KO and MB231-myrAKT transcriptomic profiles. Interestingly, the genes differentially regulated in GPS2KO cells in an AKT-independent fashion are enriched for targets of transcriptional regulation by Myc. Myc signaling may be elevated in absence of GPS2 due to a number of reasons, including (i) altered Myc transcriptional activity possibly through K63 ubiquitination (Adhikary et al., 2005), (ii) protein stabilization through modulation of Myc ubiquitination (Kim et al., 2017; Chen et al., 2019) and/or active STK38 signaling (Bisikirska et al., 2013), and/or (iii) changes in Myc gene expression, as previously reported for an handful of ERα target genes (Cheng and Kao, 2009). Further investigations will be required to address these alternative hypotheses, together with assessment of whether combined inhibition of AKT and Myc activity is more effective in inhibiting the growth of GPS2-null or mutated cells. Moreover, future studies confirming the extent of metabolic reprogramming associated with the loss/downregulation of GPS2 across different cancer types may also suggest alternative combination therapies that exploit specific metabolic vulnerabilities. Based on the characterization of various tissue-specific mouse models and the current knowledge of GPS2 role in supporting mitochondrial gene expression and mitochondria-nuclear communication (Cardamone et al., 2018; English et al., 2020), we would expect the loss or downregulation of GPS2 to correlate with altered lipid metabolism, reduced mitochondrial content and increased dependency on aerobic glycolysis. While the MDA-MB231-GPS2KO model is not ideal for investigating these predictions as TNBC cells are already characterized by high glycolytic activity and low mitochondrial respiration (Lanning et al., 2017; Guha et al., 2018; Sun et al., 2020), we did observe significant changes in the expression of key mitochondrial proteins suggesting that the loss of GPS2 could in fact be contributing to promote the metabolic reprogramming of rapidly growing cancer cells.

Another question raised by our experimental data is the extent to which GPS2 depletion leads to full activation of the signaling pathways downstream of AKT. Proteomic and phosphoproteomic profiling of MB231-GPS2KO cells indicated that upregulated proteins were enriched for terms associated with IGF/AKT/mTOR signaling pathway and for substrates of AKT-mediated phosphorylation. mTORC targets were also significantly enriched, even though some appeared downregulated rather than upregulated as one would expect downstream of PI3K/AKT signaling. Further studies will be required to validate individual targets and investigate this apparent paradox. One intriguing possibility is that GPS2-mediated regulation of AKT ubiquitination may be contributing to regulate the activation of a specific subset of downstream signaling effectors. A regulatory strategy specific for the modulation of AKT activity toward distinct downstream pathways would be consistent mTORC2-mediated activation of AKT specifically affecting lipid producing pathways via phosphorylation of ACLY, but not other downstream effectors (Martinez Calejman et al., 2020). However, it is also possible that there is a yet to be uncovered role for ubiquitination in regulating the activity of different AKT isoforms, which have been associated with opposing effects on breast cancer cell migration and invasion (Maroulakou et al., 2007; Chin and Toker, 2011; Chen et al., 2020), or in modulating AKT activation in specific subcellular locations (Sugiyama et al., 2019). Either way, the results of this study indicate that modulation of non-proteolytic ubiquitination provides yet another level of regulation to the PI3K/AKT signaling pathway and suggests that alterations to GPS2 and possibly other players in this regulatory circuit should be considered when assessing responsiveness to PI3K/AKT inhibitor treatments.

## Data Availability Statement

The datasets generated for this study are available in Gene Expression Omnibus under accession number: GSE161283, https://www.ncbi.nlm.nih.gov/geo/query/acc.cgi?acc=GSE161283.

## Ethics Statement

The animal study was reviewed and approved by Boston University IACUC.

## Author Contributions

SC, MC, and VP conceived and planned the experiments, interpreted the results, and wrote the manuscript with help and critical feedback from all other authors. SC and ES carried out both cell-based and animal-based experiments with assistance from AT-L and MC. SC, YG, and JK carried out the omics profiling experiments. BB, IT, and SM performed the analysis of omics data. All authors contributed to the article and approved the submitted version.

## Conflict of Interest

The authors declare that the research was conducted in the absence of any commercial or financial relationships that could be construed as a potential conflict of interest.

## Supplementary Material

Supplemental Figure 1 **(A)** Schema of human *Gps2* locus with showing sites targeted by sgRNAs. Two independent sets of sgRNAs targeting exon2 and exon6 were used to generate KO1 and KO2 lines. **(B)** gDNA gel showing deletion of targeted fragment in MB231-GPS2KO lines. Residual amplification of the wild type locus is observed as expected in the KO cell pools. **(C)** Venn diagram of overlapping regulated genes in the two MB231-GPS2KO cell lines.

Supplemental Figure 2 **(A)** 24-h scratch assay of MB231 transiently transfected with siRNA against GPS2 or control siRNA. **(B)** 7-h scratch assay of MB231-WT and -GPS2KO cell lines. **(C)** Distribution of GPS2 expression across tumor types in TCGA PanCancer studies reported on cBioPortal.

Supplemental Figure 3 **(A)** Ten most significant pathways associated with all differentially expressed proteins in MB231-GPS2KO based on the HumanCyc Encyclopedia of Human Genes and Metabolism database (EnrichR). Bars represent Adjusted p value as indicated in parenthesis. **(B)** Ten most significant GO cellular terms and pathways (BioCarta database, EnrichR) associated with upregulated MB231-GPS2KO proteins. Bars represent Adjusted *p* value as indicated in parenthesis. **(C)** Ten most significant GO cellular terms and pathways (BioCarta database, EnrichR) associated with downregulated MB231-GPS2KO proteins Bars represent Adjusted p value as indicated in parenthesis. **(D)** PCA plot of MB231-WT and -GPS2KO phosphosites not normalized to proteomic data.

Supplemental Figure 4 **(A)** IP/WB of poly-ubiquitinated AKT in MB231-WT and MB231-KO cells. **(B)** IP/WB of AKT1-FLAG in MB231-myrAKT cells. **(C)** Volcano plot of DEG in MB231-WT vs MB231-myrAKT (FDR ≤ 0.001, FC ≥ 2). **(D)** Volcano plot of DEG in MB231-GP2KO vs MB231-GPS2KO treated with 10 μM MK2206 for 4 h prior to collection (FDR ≤ 0.001, FC ≥ 2). **(E)** WB of pAKT and total AKT in MB231-WT cells treated with EGF (10 ng/ml, 5 min) or MK2206 (10 μM, 4 h) as indicated.

Supplemental Figure 5 **(A)** WB of GPS2, pAKT S473 and total AKT levels in WCL of MB468-WT and MB468-GPS2KO cells. **(B)** Signature projection of MB231-GPS2KO downregulated genes across TCGA PAM50 subtypes. **(C)** Kaplan–Meier survival plot (KMplot.com) of breast cancer according to GPS2 expression. Gene expression data and relapse free and overall survival information are downloaded from GEO (Affymetrix microarrays only), EGA and TCGA (Györffy et al., 2010).
