# Supplementary figures and images for: Loss of G-Protein Pathway Suppressor 2 Promotes Tumor Growth Through Activation of AKT Signaling
Source: Front Cell Dev Biol. 2021 Jan 7;8:608044. doi: 10.3389/fcell.2020.608044 (PMC7817781; doi:10.3389/fcell.2020.608044)

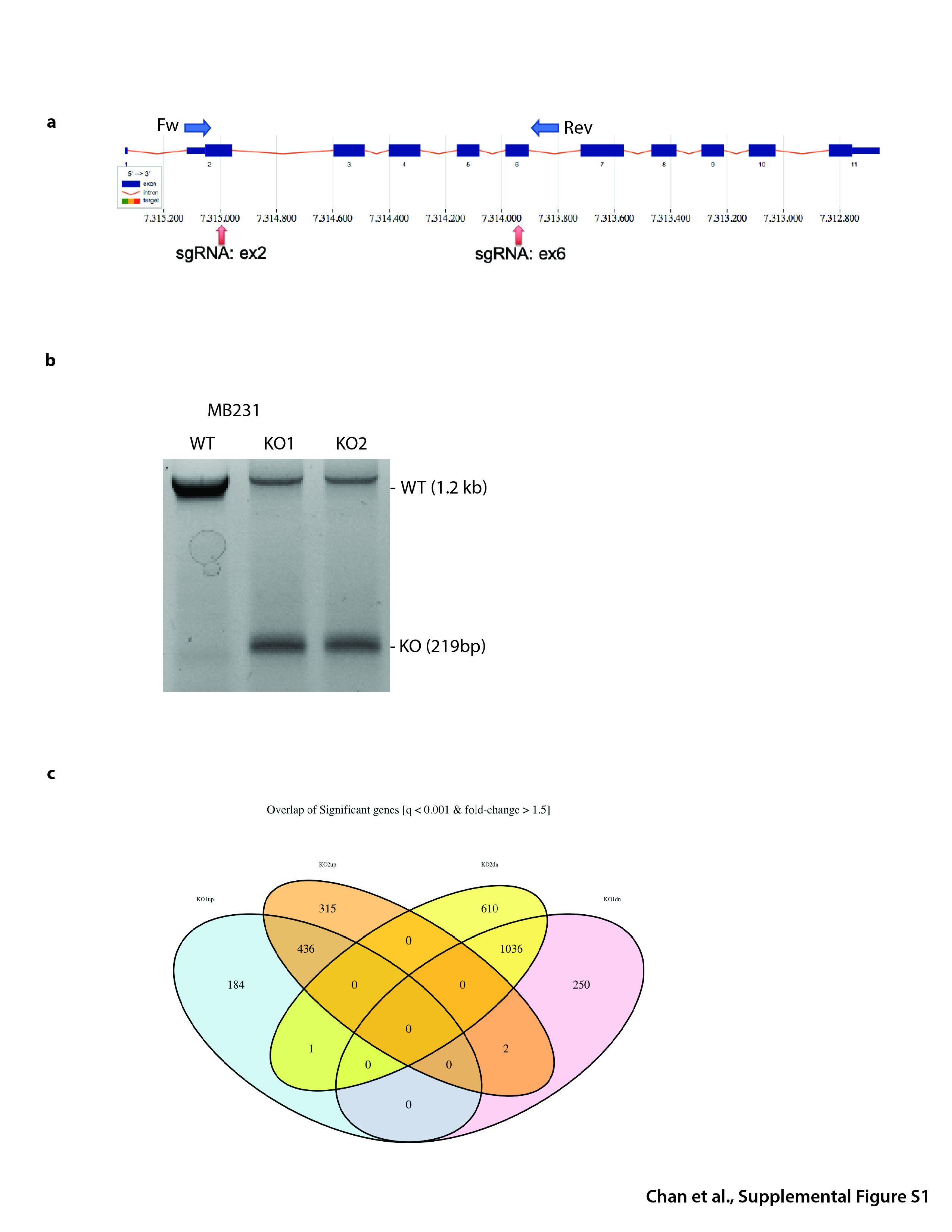

Supplement: Supplemental Figure 1 — (A) Schema of human Gps2 locus with showing sites targeted by sgRNAs. Two independent sets of sgRNAs targeting exon2 and exon6 were used to generate KO1 and KO2 lines. (B) gDNA gel showing deletion of targeted fragment in MB231-GPS2KO lines. Residual amplification of the wild type locus is observed as expected in the KO cell pools. (C) Venn diagram of overlapping regulated genes in the two MB231-GPS2KO cell lines. [file Image_1.jpg]

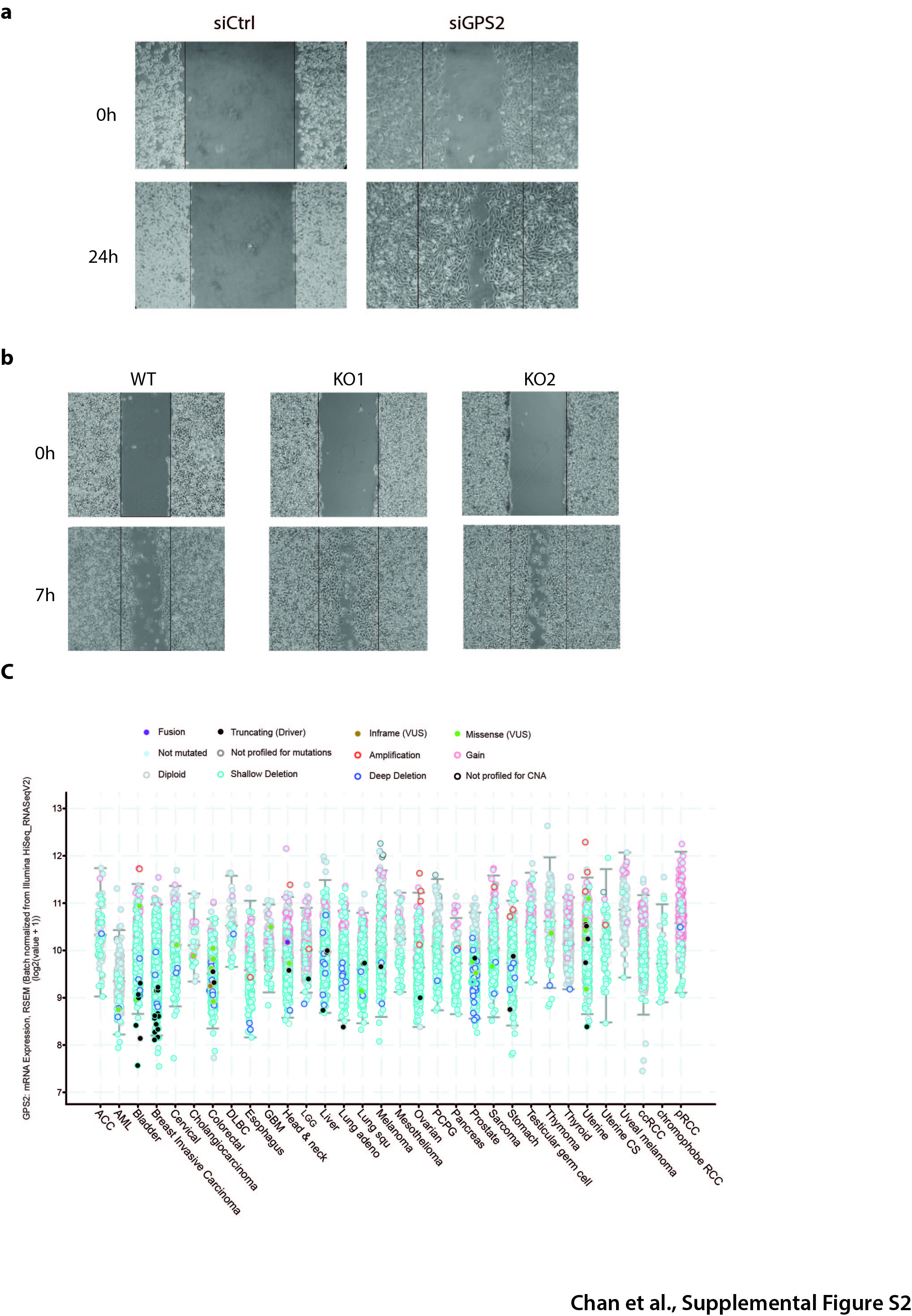

Supplement: Supplemental Figure 2 — (A) 24-h scratch assay of MB231 transiently transfected with siRNA against GPS2 or control siRNA. (B) 7-h scratch assay of MB231-WT and -GPS2KO cell lines. (C) Distribution of GPS2 expression across tumor types in TCGA PanCancer studies reported on cBioPortal. [file Image_2.jpg]

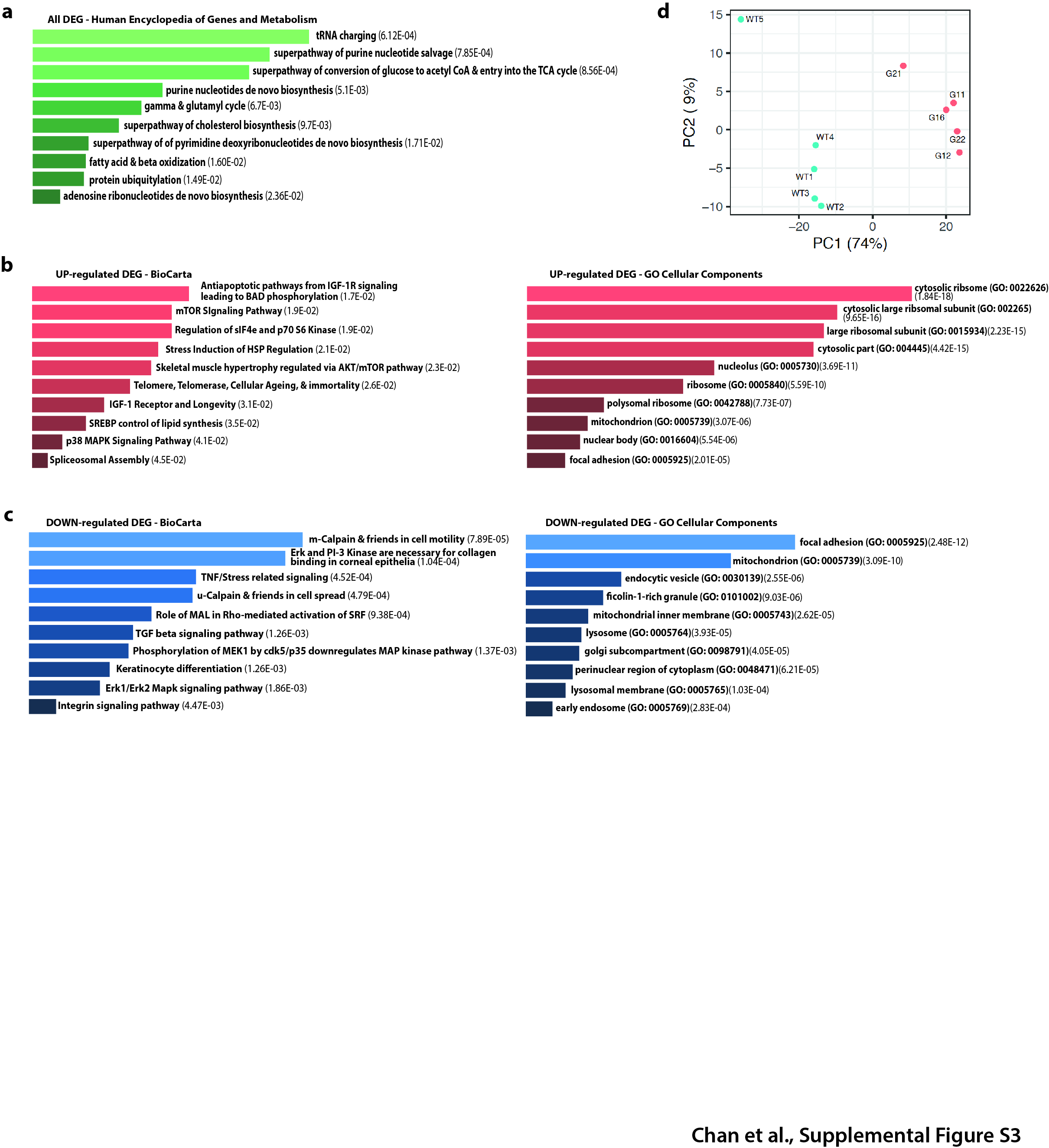

Supplement: Supplemental Figure 3 — (A) Ten most significant pathways associated with all differentially expressed proteins in MB231-GPS2KO based on the HumanCyc Encyclopedia of Human Genes and Metabolism database (EnrichR). Bars represent Adjusted p value as indicated in parenthesis. (B) Ten most significant GO cellular terms and pathways (BioCarta database, EnrichR) associated with upregulated MB231-GPS2KO proteins. Bars represent Adjusted p value as indicated in parenthesis. (C) Ten most significant GO cellular terms and pathways (BioCarta database, EnrichR) associated with downregulated MB231-GPS2KO proteins Bars represent Adjusted p value as indicated in parenthesis. (D) PCA plot of MB231-WT and -GPS2KO phosphosites not normalized to proteomic data. [file Image_3.jpg]

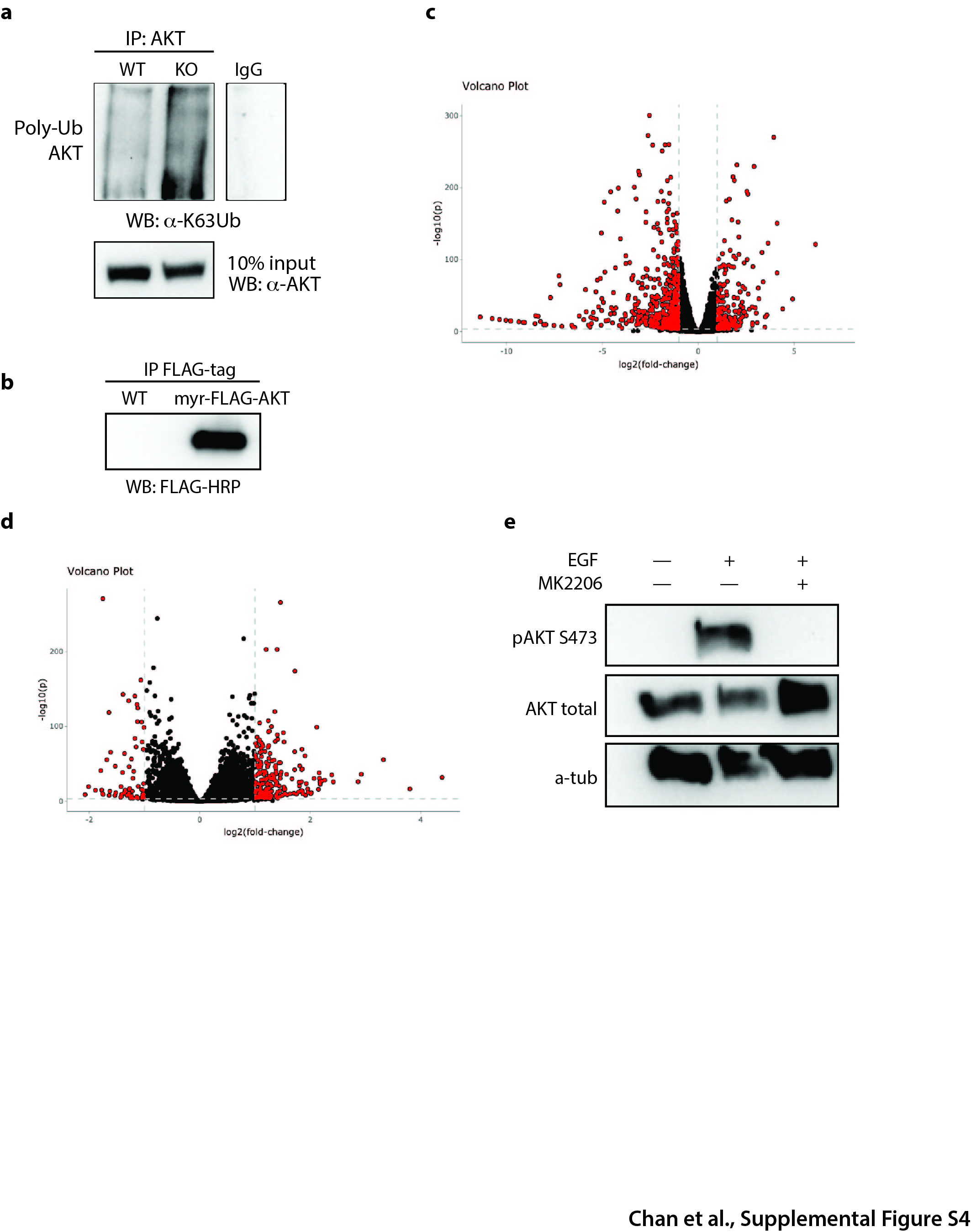

Supplement: Supplemental Figure 4 — (A) IP/WB of poly-ubiquitinated AKT in MB231-WT and MB231-KO cells. (B) IP/WB of AKT1-FLAG in MB231-myrAKT cells. (C) Volcano plot of DEG in MB231-WT vs MB231-myrAKT (FDR ≤ 0.001, FC ≥ 2). (D) Volcano plot of DEG in MB231-GP2KO vs MB231-GPS2KO treated with 10 μM MK2206 for 4 h prior to collection (FDR ≤ 0.001, FC ≥ 2). (E) WB of pAKT and total AKT in MB231-WT cells treated with EGF (10 ng/ml, 5 min) or MK2206 (10 μM, 4 h) as indicated. [file Image_4.jpg]

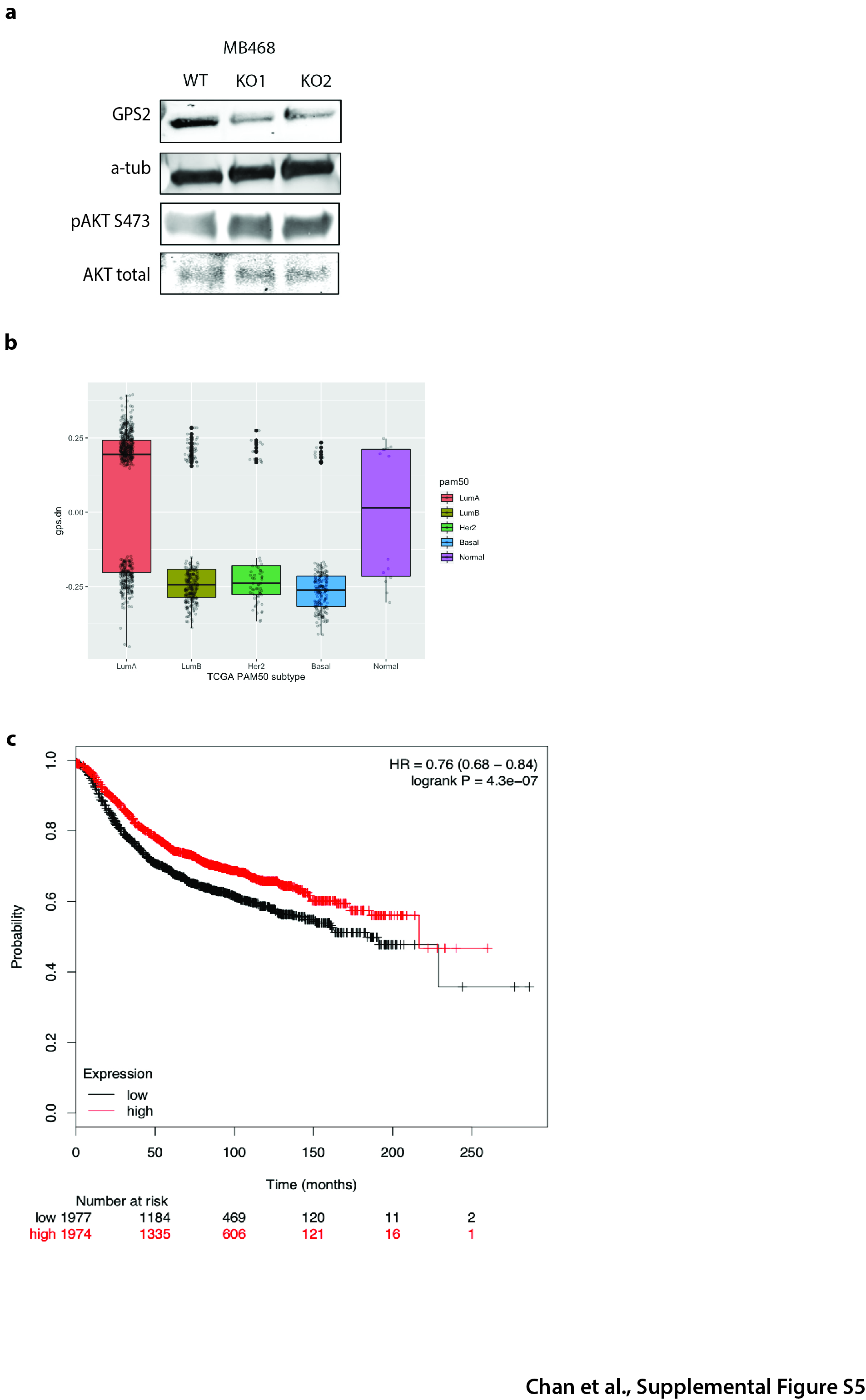

Supplement: Supplemental Figure 5 — (A) WB of GPS2, pAKT S473 and total AKT levels in WCL of MB468-WT and MB468-GPS2KO cells. (B) Signature projection of MB231-GPS2KO downregulated genes across TCGA PAM50 subtypes. (C) Kaplan–Meier survival plot (KMplot.com) of breast cancer according to GPS2 expression. Gene expression data and relapse free and overall survival information are downloaded from GEO (Affymetrix microarrays only), EGA and TCGA (Györffy et al., 2010). [file Image_5.jpg]
